# Dr. William B. Pierce

**Published:** 1913-06

**Authors:** 


Dr. William B. Pierce of Buffalo, died at his summer home in Fort Erie, Can., May 22, aged 77. He was born in Friendship, N. Y. His name is not listed in either Polk or the State Directory.

